# Natural variation in gene expression in the early development of dauer larvae of *Caenorhabditis elegans*

**DOI:** 10.1186/1471-2164-10-325

**Published:** 2009-07-18

**Authors:** Simon C Harvey, Gary LA Barker, Alison Shorto, Mark E Viney

**Affiliations:** 1 School of Biological Sciences, University of Bristol, Woodland Road, Bristol, BS8 1UG, UK; 2 Department of Geographical and Life Sciences, Canterbury Christ Church University, North Holmes Road, Canterbury, CT1 1QU, UK

## Abstract

**Background:**

The free-living nematode *Caenorhabditis elegans *makes a developmental decision based on environmental conditions: larvae either arrest as dauer larva, or continue development into reproductive adults. There is natural variation among *C. elegans *lines in the sensitivity of this decision to environmental conditions; that is, there is variation in the phenotypic plasticity of dauer larva development. We hypothesised that these differences may be transcriptionally controlled in early stage larvae. We investigated this by microarray analysis of different *C. elegans *lines under different environmental conditions, specifically the presence and absence of dauer larva-inducing pheromone.

**Results:**

There were substantial transcriptional differences between four *C. elegans *lines under the same environmental conditions. The expression of approximately 2,000 genes differed between genetically different lines, with each line showing a largely line-specific transcriptional profile. The expression of genes that are markers of larval moulting suggested that the lines may be developing at different rates. The expression of a total of 89 genes was putatively affected by dauer larva or non-dauer larva-inducing conditions. Among the upstream regions of these genes there was an over-representation of DAF-16-binding motifs.

**Conclusion:**

Under the same environmental conditions genetically different lines of *C. elegans *had substantial transcriptional differences. This variation may be due to differences in the developmental rates of the lines. Different environmental conditions had a rather smaller effect on transcription. The preponderance of DAF-16-binding motifs upstream of these genes was consistent with these genes playing a key role in the decision between development into dauer or into non-dauer larvae. There was little overlap between the genes whose expression was affected by environmental conditions and previously identified loci involved in the plasticity of dauer larva development.

## Background

Developmental decisions and processes can be controlled transcriptionally. The free-living nematode *Caenorhabditis elegans *makes a developmental decision between different larval fates. This decision is based on the 'suitability' of the environment for growth and reproduction. Under 'favourable' conditions, second stage larvae (L2) develop *via *two larval stages (L3, L4) into reproductive adults [1,2]. However, under 'unfavourable' conditions, L2s form a developmentally arrested L3 stage, the so-called dauer larva. Dauer larvae are environmentally resistant, have a specialised metabolism and are comparatively long-lived [2]. Overall, dauer larvae are transcriptionally repressed compared with actively growing, non-dauer larva stages [3]. However, the expression of some genes is comparatively enhanced in, or specific to, dauer larvae [3-6], showing that this transcriptional repression does not apply to all genes. If environmental conditions 'improve' then dauer larvae resume their development *via *the L4 stage. Thus, the decision whether to develop into dauer larvae or into 'normal', non-dauer larvae is environmentally determined. The dauer or non-dauer developmental programme will, at least in part, be executed by transcriptional control.

The features of the environment that are used by larvae making this developmental decision are the concentration of food, the concentration of dauer pheromone and temperature. Dauer pheromone is a cue produced by all worms that acts as a measure of con-specific population density [7] and appears to consist of at least three related molecules [8,9]. Conditions that favour the development of dauer larvae are a low concentration of food and a high concentration of dauer pheromone (*i.e*. a high conspecific population density). Conversely, conditions that favour the development of non-dauer larva development are a high concentration of food and a low concentration of dauer pheromone. Higher temperatures favour the development of dauer larvae [1].

There has been extensive investigation into the genetic and molecular genetic control of the development of dauer larvae, which is known to be controlled by a TGF-β-like pathway, an insulin-like pathway and a guanyl cyclase pathway [1,2]. There have been a number of studies that have compared gene expression in dauer larvae with other life-cycle stages [5], compared L2, L3 and dauer larvae of wild type and mutant lines [6,10] or determined how gene expression changes during entry into the dauer larva stage [11]. These studies have found large differences in the transcriptional profiles of these stages, fully consistent with the different morphology and physiology of dauer larvae. Genes involved in the insulin-like pathway, particularly the FOXO-family transcription factor *daf-16*, have been shown to be key in the generation of these transcriptional differences [12-14]. However, these studies have not investigated variation in gene expression between isolates nor the very early stages of the dauer/non-dauer larva decision. At these early stages it can be envisaged that there may be small differences that initiate subsequent larger transcriptional changes. In this sense, previous studies have investigated changes in gene expression that are associated with dauer development rather than the genes that are involved in making the decision between dauer and non-dauer larval development.

The natural history of *C. elegans *is still poorly understood. However, individual *C. elegans *are most often isolated from the wild as dauer larvae, rather than as reproducing adults [15]. This observation suggests two things: firstly, that the dauer larva morph is of central importance in the natural history of *C. elegans *and, secondly, that dauer larvae and the developmental decision whether or not develop into dauer larvae is likely to be under strong natural selection. Previously we have compared the plasticity of dauer larva development of different lines of *C. elegans *[16,17]. Plasticity is a measure of the sensitivity of lines to different environmental conditions, with this sensitivity measured as the difference in the proportion of larvae that develop into dauer larvae between two or more different environments. We have found that lines of *C. elegans *differ in their plasticity of dauer larva development [16,17]. For example, over a range of concentrations of dauer pheromone, in some lines, only a few individuals will develop as dauer larvae (*i.e*. low plasticity lines); in other lines, the proportion of individuals that develop as dauer larvae will increase rapidly with the concentration of dauer pheromone (*i.e*. high plasticity lines).

Genetic analysis of variation in the plasticity of dauer larvae formation has identified a number of quantitative trait loci (QTL) that control it [17]. Given that transcriptional differences are also likely to be involved in dauer larva development we wished to determine whether such transcriptional differences originated from these QTL regions. More particularly, we hypothesised that inter-line differences in the phenotypic plasticity of dauer larvae development of *C. elegans *is due to inter-line transcriptional differences. To investigate this we have investigated the transcriptional profiles of *C. elegans *lines with different phenotypic plasticities of dauer larva development. We further hypothesised that the different dauer larva development plasticities were most likely to be due to interline differences in the 'decision' and early initiation of development into non-dauer or dauer larvae. For this reason we compared the transcriptional profile of early stage larvae exposed to dauer larva or 'normal', non-dauer larva-inducing conditions.

## Methods

### Worms

*C. elegans *lines N2 and DR1350 and two recombinant inbred lines (RILs), RIL-14 and RIL-17 were used. N2 and DR1350 were obtained from the *Caenorhabditis *Genetics Center. The RILs were previously generated by taking F1 progeny of a N2 × DR1350 cross which were then allowed to self-fertilise for at least 30 generations, as previously described [16,17]. All lines were maintained on standard NGM plates [18] with an *Escherichia coli *OP50 food source.

Previously, the phenotypic plasticity of dauer larva development of these lines was determined [16,17,19]. N2 has a higher plasticity (0.39 and 0.27 difference in the proportion of dauer larvae that develop in response to a change in pheromone and food concentration, respectively [16,17]) than DR3150 (0.23 and 0.15 difference in the proportion of dauer larvae that develop in response to a change in pheromone and food concentration, respectively [16,17]). Similarly, RIL-17 has a higher plasticity (0.53 and 0.50 to a change in pheromone and food concentration, respectively) than RIL-14 (0.07 and 0.05 to a change in pheromone and food concentration, respectively [16,17]).

### Larval growth

These analyses were undertaken in two experiments. In experiment one N2 and DR1350 were analysed; in experiment two RIL-14 and RIL-17 were analysed. For each of the four lines, L1s were grown in 'normal', non-dauer larva-inducing or in dauer larva-inducing conditions until they were halfway through the L2 stage, at which point the larvae were harvested for the preparation of RNA for use with *C. elegans *whole genome microarrays.

For each replicate of each line, 30,000 age synchronous eggs were liberated from hypochlorite treated gravid hermaphrodites [18] that had been grown under standard conditions. The liberated eggs were maintained overnight in 5 mL S medium [18] in the absence of food, in a 50 mL tube stoppered with cotton wool at 25°C whilst being shaken at 200 rpm. Under these conditions, the eggs hatched into L1s, but did not develop further. An *E. coli *OP50 food source was then added to achieve a final concentration of 20 mg/mL; this was the 'normal', non-dauer larva-inducing conditions. We had previously determined that this concentration of food was sufficient for L1s to grow to adulthood and, thus, that these are 'normal' and non-starvation conditions (data not shown). The dauer larva-inducing conditions were the same, but with the addition of 450 μL of dauer pheromone to each tube. All cultures were maintained for a further 8.5 hours, at 25°C while being shaken at 200 rpm; at this time the larvae are temporally half-way through the L2 stage. Development was confirmed by microscopic analysis of the larvae in pilot experiments (data not shown). At this point 0.5 mL of each culture was removed and maintained in the same conditions for a further two days to confirm the phenotype (*i.e*. development into dauer larvae or into adults, thus a measure of the plasticity of dauer larva development) of the worms grown under these two conditions. The remainder of each sample was centrifuged for two minutes at 850 ***g***, the supernatant removed and the worms resuspended in M9 buffer [18]; this was repeated three times. Worms were then centrifuged at 850 ***g ***for 6 minutes at 4°C on a 60% v/v: 40% v/v Percoll gradient in M9 to remove residual *E. coli *OP50, the worms removed from the interface, washed three further times by sedimentation and resuspension in M9, before being finally resuspended in a small volume of M9 to which an equal volume of Tri reagent (Sigma-Aldrich) was added. Samples were then snap frozen in liquid nitrogen and stored at -80°C. RNA was extracted following the manufacturer's instructions. For each line of *C. elegans *there were three biological replicates for each of the two environmental conditions. Dauer pheromone was prepared from liquid cultures of N2 as previously described [20]. All the pheromone used for these studies was from the same batch. The *E. coli *OP50 food source was prepared from shaken liquid cultures grown overnight in LB media at 37°C, after which the bacterial suspension was centrifuged for 15 minutes at 5,500 ***g***, and the supernatant removed, with the pellet of bacteria resuspended in M9 at a concentration of 0.2 g/mL.

### Microarray analysis

RNA samples for microarrays were processed and microarray hybridisation was performed by the Gene Microarray centre of the Institute of Child Health, University College London. RNA integrity was checked using a Bioanalyser 2100 (Agilent). 5 μg of total RNA from each sample was converted to cDNA using an oligo(dT) primer and a microarray cDNA synthesis kit (Roche). The cDNA was then labelled using an Affymetrix labelling kit and the product fragmented and hybridised to *C. elegans *GeneChip genome arrays (Affymetrix) following the manufacturer's instructions. These chips represent 22,500 transcripts of the expressed *C. elegans *genome based on the December 2005 genome sequence and GenBank release 121. The microarray data reported here have been deposited at ArrayExpress http://www.ebi.ac.uk/microarray-as/aer/entry with accession number E-MEXP-1808 and E-MEXP-1810 and have been submitted to WormBase http://www.wormbase.org.

### Data analysis

Data were analysed separately using a series of custom PERL scripts. For within-chip normalisation, the log_2 _transformed intensities for individual features were scaled by subtracting the mean chip log_2 _intensity value, and centred by dividing by the chip standard deviation. Each experiment was analysed separately, with between-chip normalisation and distribution normalisation applied across all chip data within that experiment as described elsewhere [21]. Briefly, to do this, the values for each chip were ranked from lowest to highest and the mean value for each rank calculated across all chips. Each chip value was then replaced by the corresponding mean value for its rank. This results in datasets where chips have identical distributions but have the granularity of a full dynamic range of expression values preserved. The normalised data were analysed using a two-way ANOVA, testing for each gene the effects of LINE (N2, DR1350, RIL-14, RIL-17), TREATMENT (non-dauer *vs *dauer larva-inducing) and the LINE × TREATMENT interaction, using a published PERL script [22]. In this way the *F*_1,8 _values (and hence *p *values) for the effects of LINE, TREATMENT and LINE × TREATMENT for each gene were determined.

To validate the microarray results we used semi-quantitative RT-PCR to analyse the expression of 15 genes in N2 and DR1350. To do this 0.5 μg of total RNA was transcribed into cDNA and this template used in PCR reactions, all as previously described [23] for 30 cycles for the following genes: R57.2, W04G3.1, F52B11.3, W08F4.6, T12B5.11, M03F4.5, K07E1.1, C55B7.4, M03A1.3, F58E10.4, T03G6.1, M03A1.3, M05D6.7, F44E2.4 and ZK899.4. Primers were designed to amplify across an intron-containing region to control for any gDNA contamination. In these analyses we compared the concentration of the resulting amplicon from N2 and from DR1350 in three replicate RNA samples from each line.

Gene annotation, including gene ontology (GO) annotation was taken from WormBase release WS180 (7^th ^September 2007) [24]. Genes of interest resulting from these experiments were compared using a Chi-squared test, using GeneList [25], to existing groups of genes lists, defined by GeneList from user entries, genomic annotation sources including GO and other functional categories from WormBase, the Kyoto Encyclopaedia of Genes and Genomes (KEGG) and the *C. elegans *gene expression topomap. This determined the probability that the occurrence of the selected 'query' genes within each of the 'target' groups of genes was random. To identify potential regulatory regions in sequences upstream of genes of interest, 500 bp of sequence immediately 5' of each gene (WormBase release WS180) was analysed using BioProspector [26], with default settings and an 8 bp analysis window. We then compared the frequency of the occurrence of candidate regulatory regions in 500 bp 5' of the genes of interest with the frequency of the occurrence of that regulatory region in 500 bp 5' of all *C. elegans *genes. We used a hypergeometric test to determine the probability that the observed distribution of such regions among genes of interest was different to the distribution among all genes.

## Results and discussion

### Microarray quality control

Analysis and quality control of the microarray data showed that data were available for all replicates and treatments for 15,792 genes from experiment one (N2 and DR1350) and for 14,759 genes from experiment two (RIL-14 and RIL-17). Of the 15 genes analysed by semi-quantitative PCR, 13 could be successfully amplified and the expression of 9 (69%) was concordant with the microarray data (R57.2, W04G3.1, F52B11.3, W08F4.6, M03F4.5, K07E1.1, F58E10.4, T03G6.1 and M05D6.7).

### Inter-line differences in gene expression

#### Large differences in gene expression exist between isolates

There were substantial differences in gene expression between the different *C. elegans *lines within the same environmental conditions. There were 2,920 genes whose expression differed significantly (LINE*p *< 0.001) between N2 and DR1350 (1,338 DR1350 > N2; 1,582 N2 > DR1350) and 221 whose expression differed significantly (LINE*p *< 0.001) between RIL-14 and RIL-17 (136 RIL-14 > RIL-17; 85 RIL-17 > RIL-14). These genes are listed in additional file 1. It is of interest that there are more genes whose expression is significantly different between N2 and DR1350 than between RIL 14 and RIL-17, because N2 and DR1350 are genetically more distinct from each other, compared with the two RILs which are F1 progeny of a N2 × DR1350 cross. This supports the idea that these gross transcriptional differences are genetically determined. Another comparison of two *C. elegans *lines across all developmental stages also found substantial inter-line transcriptional differences [27]. Analysis of gene transcription in the yeast *Saccharomyces cerevisiae *has shown that a naturally occurring single frameshift mutation controls 45% (103 genes) of the transcriptional differences between two lines [28]. Thus, small genetic differences can have substantial transcriptional consequences. Other analyses of *S. cerevisiae *have shown inter-line differences in gene expression are controlled by *trans*-acting loci, that are not necessarily transcription factors [29]. Further analysis of yeast has shown that there is also significant inter-cell, intra-line, differences in expression (*i.e*. 'noise') which is genetically controlled [30].

#### Inter-line differences are non-random

We compared the occurrence of genes significantly differently expressed in N2 and DR1350 to previously prepared gene lists [25]. This showed that N2 and DR1350 had significantly different transcriptional profiles (Table 1). That is, genes with comparatively significantly greater expression in N2 are significantly over represented in certain gene lists and that this representation is different for genes with comparatively significantly greater expression in DR1350. These differences are striking. For example, in DR3150 there is significantly greater expression of mount 14 and histone genes *etc*. compared with N2. Analogously, in N2 there is significantly greater expression of mount 6 and 2 genes *etc*., compared with DR1350. RIL-14 and RIL-17 also appear to have different transcriptional profiles (Table 2), though there are fewer identifiable categories because of the smaller differences between the transcription of these lines. That the transcriptional differences that occur between the lines are non-random suggests that the lines are following different transcriptional profiles. Analogously with the studies of yeast, these differences are likely to be controlled by relatively few loci, that may or may not be transcription factors [28,29]. These small number of loci may well act early in development (*e.g*. during the L1 stage), but their transcriptional effect is amplified as development proceeds.

**Table 1:** N2 and DR1350 inter-line differences in gene expression.

Gene list	Query/List	*p *value
		

**Up in DR1350 compared with N2**		

Mount 14	147/352	< 1.880e-105
Histones	32/73	< 6.591e-23
Mount 1	161/1698	< 2.754e-20
Mount 36	9/10	< 4.845e-11
Mount 32	13/24	< 1.222e-10
Heat shock	14/33	< 1.533e-09
Cell adhesion	13/45	< 2.281e-06
Mount 16	29/223	< 1.009e-05
Cell structure	28/218	< 2.133e-05
Collagen	24/179	< 6.758e-05
Amino acid metabolism	16/104	< 7.338e-04
MSP	10/43	< 7.646e-04
Carbohydrate metabolism	16/120	< 0.005

**Up in N2 compared with DR1350**		

Mount 6	168/892	< 2.303e-35
Mount 2	186/1418	< 3.745e-19
Mount 8	122/803	< 7.331e-17
Mount 5	124/915	< 3.251e-13
Biosynthesis	78/475	< 2.733e-12
RNA pol II transcription	61/370	< 1.483e-09
Mount 1	180/1698	< 2.261e-09
Mount 20	36/159	< 2.543e-09
Germ line-enriched	73/507	< 1.089e-08
Mount 11	79/576	< 2.035e-08
Protein expression	59/380	< 3.368e-08
Mount 15	43/247	< 2.352e-07
Mitochondrial	32/164	< 1.432e-06
RNA binding	35/209	< 1.736e-05
Dauer	10/22	< 1.952e-05
Mount 24	23/133	< 0.001
tRNA synthetase	9/30	< 0.005
Mount 31	8/25	< 0.007

The gene lists that are significantly over represented among genes whose expression is significantly different between N2 and DR1350. Query/List shows the number of genes whose expression is significantly different between N2 and DR1350/the total number of genes within the gene list. *p *value is the probability that the occurrence of the number of query genes in the gene list is random, with this Holm-Bonferroni corrected for multiple testing. MSP – major sperm protein. Expression mounts are as defined by [40].

**Table 2:** RIL-14 and RIL-17 inter-line differences in gene expression.

Gene list	Query/List	*p *value
		

**Up in RIL-14 compared with RIL-17**		

Mount 14	42/352	<2.320e-44
Mount 1	26/1698	<3.928e-05
Cell Adhesion	4/45	<0.008

**Up in RIL-17 compared with RIL-14**		

Mount 8	15/803	<6.667e-06
Mount 25	4/101	<0.039

The gene lists that are significantly over represented among genes whose expression is significantly different between RIL-14 and RIL-17. Query/List shows the number of genes whose expression is significantly different between RIL-14 and RIL-17/the total number of genes within the gene list. *p *value is the probability that the occurrence of the number of query genes in the gene list is random, with this Holm-Bonferroni corrected for multiple testing. Expression mounts are as defined by [40].

#### Measured inter-line transcriptional differences are not due to sequence variation

There are significant genome sequence differences between N2 and DR1350 [17] which may have the potential to affect measurement of gene expression because the microarray is based on the N2 genome. Thus, transcripts of DR1350, RIL-14 and RIL-17 may hybridise less strongly to the microarray compared with N2, thereby generating false measures of different gene expression. However, we believe, for two reasons, that this situation did not occur. Firstly, there was no gross bias towards N2 in the measured transcriptional differences of N2 (1,582 genes expressed more, compared with DR1350) compared with DR1350 (1,338 genes expressed more, compared with N2). Secondly, the inter-genome differences between N2 and DR1350 predominantly occur on chromosomes I, II, III and X; chromosomes IV and V appear to be virtually identical [17]. The chromosomal distribution of genes whose expression differs significantly between N2 and DR1350 occurs on all six chromosomes and, indeed, appears to mirror the gene densities of each chromosome [31] (data not shown). For these two reasons we consider that the use of a N2 microarray to analyse gene expression in four different *C. elegans *lines has not resulted in the generation of erroneous data.

#### Inter-line differences may be due to differences in developmental rate

The analyses above suggest that these *C. elegans *lines are following different transcriptional profiles. An alternative possibility is that they are actually following the same transcriptional profile, but that the lines are moving through this at different rates. In support of this, for example, DR1350 reaches sexual maturity approximately 2–3 hours earlier than N2 at 25°C [32], suggesting that the developmental progression of DR1350 is quicker compared with N2.

To investigate this further we compared the expression of 10 genes that are key indicators of the early stages of the preparation for the L2 – L3 moult. These 10 genes are known to be involved in the moulting process and there is an increase in the abundance of their transcripts from the mid-L2 stage [33-35]. The worms used in our experiments were at the mid-L2 stage (defined temporally) and therefore it would be expected that the expression of these 10 genes would be low. The expression of 9 of these genes (*mlt-8, 9, 11, sqt-1, 3, dpy-7, 9 *(*p *< 0.001); *daf-9, mlt-10 *(*p *< 0.05)) was significantly greater in DR1350 compared with N2; one was not (*col-12*, *p *> 0.05). This suggests that DR1350 is more developmentally advanced (*i.e*. closer to the moult into L3) compared with N2. Similarly, in RIL-14 and RIL-17 the expression of 6 of these genes (*mlt-8, 9 *(*p *< 0.001); *mlt-10, sqt-1, dpy-7, 9 *(*p *< 0.05)) were significantly greater in RIL-14 compared with RIL-17; four were not (*daf-9*, *mlt-11, sqt-3, col-12 *(*p *> 0.05)). Together, these data strongly suggest that DR1350 and RIL-14 are more developmentally advanced compared with N2 and RIL-17, respectively, and therefore that the interline differences in transcription that we have observed may be due to differences in developmental progression. The changes in gene expression over the *C. elegans *life-cycle are therefore likely to be even more complex than those identified so far [27].

It is notable that DR1350 and RIL-14 have lower phenotypic plasticities of dauer larva formation compared with N2 and RIL-17. It is therefore possible that the lines have different sensitivity to dauer larva-inducing conditions [16]. If the lines have different rates of developmental progression then different sensitivities to environmental conditions may be a consequence of this. Thus, comparatively faster development in DR1350 and RIL-14 may result in a shorter time when larvae are sensitive to environmental conditions, compared with N2 and RIL-17, which is manifest as a reduced sensitivity to these conditions.

### Gene expression in different environments

#### 'Normal' and dauer larva-inducing conditions affect the expression of few genes

**Figure 1:**
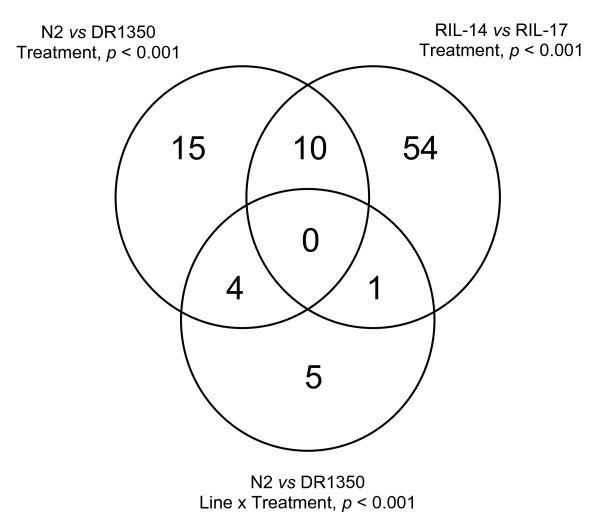
**Gene expression in different environments for the four lines**. Venn diagram showing the distribution of the number of genes whose expression significantly differs between dauer larva and non-dauer larva-inducing conditions for N2, DR1350, RIL-14 and RIL-17.

The expression of relatively few genes was different between larvae exposed to 'normal', non-dauer larva and dauer larva-inducing conditions. In N2 and DR1350, 29 genes differed significantly (TREATMENT*p *< 0.001) in their expression between these two conditions. 13 genes were expressed comparatively more in dauer larva-inducing conditions, 16 in non-dauer larva-inducing conditions. In RIL-14 and RIL-17, 65 genes differed significantly (TREATMENT*p *< 0.001) in their expression between these two conditions; 22 genes were expressed comparatively more in dauer larva-inducing conditions, 43 in non-dauer larva-inducing conditions. The expression of 10 genes was affected by environmental conditions differently between N2 and DR1350 (*i.e*. a genotype-by-environment effect) (ISOLATE × TREATMENT*p *< 0.001), with the expression of 4 of these also differing between treatments (TREATMENT*p *< 0.001), but there was no such effect for RIL-14 and RIL-17 (Figure 1).

Thus, in contrast to the inter-line transcriptional differences, different environments have rather smaller effects on gene expression. This observation is in notable contrast to an analogous study of gene expression in yeast, where comparison of two lines, in two environments found a large number of line, environment and line × environment (2,996, 3,448, 2,037 transcripts affected, respectively) effects [36]. Our findings are also in contrast to studies which have compared gene expression between dauer larvae and other stages [5] or compared gene expression in L2, L3 and dauer larvae of wild type and mutant lines [6,10], which found differences in hundreds or thousands of genes. In the development of dauer and non-dauer larvae there is an initial 'choice', followed by subsequent execution of dauer or non-dauer larval phenotypes. As such, once a developmental decision has been made, it can be envisaged that there is an increasing transcriptional divergence. The large transcriptional differences seen in other studies are likely to be the consequence of the completion of these different larval fates. We specifically sought to measure transcription early in the initiation of dauer or non-dauer larval development, and for this reason differences in the expression of comparatively few genes is what would be expected.

#### The four *C. elegans *lines have similar changes in gene expression

There was some commonality between the results from the two experiments (N2 and DR1350; RIL-14 and RIL-17) (Figure 1). This therefore suggests that despite the inter-line transcriptional differences (above) there is a core common transcriptional response of exposure to dauer or non-dauer larva-inducing conditions. From these data we therefore identified a set of 89 genes whose expression is significantly affected (TREATMENT and LINE × TREATMENT*p *< 0.001) in larvae exposed to non-dauer larva or dauer larva-inducing conditions in at least one of the experiments (Table 3).

**Table 3:** Gene expression in different environments.

Gene	LG	Start (bp)	Gene	N2 *vs *DR1350				RIL-14 *vs *RIL-17		
					
				T	L	T × L	DI	T	L	DI
*mppa-1*	I	1740648	Mitochondrial processing peptidase alpha	*			↓	**		↓
Y54E10A.16	I	3246074	-					**		↑
F27C1.10	I	5433925	-	**	**	**	↑			
T09B4.8	I	6164721	Alanine-glyoxylate aminotransferase*					**		↓
*sdha-2*	I	10683275	Succinate dehydrogenase complex, subunit A					**		↓
*prx-11*	I	12971603	Peroxisome assembly factor	**	**		↑			
W04A8.4	I	13841882	-		**			**	**	↑
C50D2.5	II	98402	-					**		↓
Y110A2AL.4	II	2839303	-	*	**	**	DR↓			
F12E12.11	II	3742459	-	**	**		↑			
*acdh-2*	II	5547461	Acyl coA dehydrogenase					**		↑
*sra-29*	II	6574325	Serpentine receptor, class A					**		↓
*icd-1*	II	6587524	Inhibitor of cell death (RNA polymerase II general transcription factor*)		**			**		↓
T02G5.2	II	7095427	-	**	**		↑			
DH11.2	II	8012740	-	**	**		↑			
*pyr-1*	II	8651059	Pyrimidine biosynthesis	*			↓	**		↓
C01G6.7	II	9291932	*acs-7 *(fatty acid coA syhthetase family)	*	**		↑	**		↑
*cyp-13A5*	II	9798193	Cytochrome P450 family					**		↑
C05C10.4	II	9931760	*pho-11 *(intestinal acid phosphatase)	*	**		↓	**		↓
B0334.3	II	11489513	Thiamine pyrophosphate-requiring enzyme*					**		↑
F01D5.1	II	13996851	Secreted surface protein*					**		↓
F58B6.1	III	1112319	-	*	**	**	DR↓	*		↓
Y54H5A.1	III	5153423	Ribosome assembly protein*		**			**		↓
*dhs-9*	III	5364479	Dehydrogenase, short chain	**			↑	*		↑
*dlc-1*	III	6462849	Dynein light chain		**			**		↓
F44B9.2	III	8022392	-					**		↓
*eif-3.D*	III	8969556	Eukaryotic initiation factor					**		↓
*iff-1*	III	9745765	Initiation factor homologue	*			↓	**		↓
*ran-1*	III	10746465	Ran GTPase orthologue		**			**		↓
T28D6.6	III	11326536	GTP-binding protein DRG1					**		↓
Y111B2A.2	III	12495966	-	*			↓	**		↓
Y37D8A.18	III	12926440	Mitochondrial ribosomal protein S10*		**			**		↓
*abce-1*	III	13153697	ABC transporter class E	**	**		↓	**	**	↓
*tag-61*	III	13463042	*ant-1.1 *(adenine nucleotide translocator)					**		↑
K02D7.1	IV	292425	Purine nucleoside phosphorylase*	*	**		↑	**		↑
C18H7.1	IV	615544	von Willebrand factor and related coagulation proteins*					**		↑
K03H6.2	IV	1510563	-	*	**	**	N2↑			
C17H12.4	IV	6798908	Carboxylesterase and related proteins*	*		**	↑	**		↑
D2096.8	IV	8377687	Nucleosome assembly protein*		**			**		↓
T20D3.3	IV	9333319	-		**			**		↓
C08F8.2	IV	11149161	Mitochondrial RNA helicase SUV3*		**			**		↓
*kin-4*	IV	11436618	Protein kinase					**		↓
F08G5.6	IV	12435797	-	**			↓	**	**	↓
*clec-186*	IV	12866085	C-type lectin	**		**	↓	*		↓
F55G11.2	IV	12967418	-	**	**		↓	*		↓
F49E11.11	IV	13059587	*scl-3 *(SCP-like extracellular protein)	**			↓	**		↓
C39E9.4	IV	13068737	*scl-6 *(SCP-like extracellular protein)	*			↓	**		↓
*ugt-22*	IV	13639234	UDP-glucuronosyl transferase	**			↓	**		↓
Y43D4A.2	IV	16744614	-	**	**		↓	*		↓
ZK550.6	IV	17260269	Peroxisomal phytanoyl-CoA hydroxylase*	**	**		↑	**		↑
C49C3.7	IV	17332528	-					**		↑
*dod-19*	V	411783	Downstream of DAF-16	*			↓	**		↓
C14C6.5	V	536629	-	**			↓			
W07B8.4	V	1130090	Cysteine proteinase Cathepsin L*	**		**	↑	*		↑
Y19D10A.9	V	2351676	*clec-29 *(C-type lectin)	**	**		↓	**		↓
F56A4.10	V	2461320	Permease of the major facilitator superfamily*	**	**		↓	**		↓
*cyp-35A5*	V	3936359	Cytochrome P450 family	*			↓	**		↓
T22F3.11	V	3612160	Permease of the major facilitator superfamily*	**			↑	**		↑
F13H6.4	V	6366416	Carboxylesterase and related proteins*	**			↓			
*ugt-41*	V	7161049	UDP-glucuronosyl transferase	*			↓	**		↓
*cyp-35A2*	V	7362298	Cytochrome P450 family	**			↓	*	**	↓
*srx-3*	V	9005518	Serpentine receptor, class X	*	**	**	N2↓			
*dhs-18*	V	11045678	Dehydrogenase, short chain	**	**		↑	*		↑
*snf-11*	V	11305047	Sodium neurotransmitter symporter family					**		↓
F35B12.4	V	11609217	Serine proteinase inhibitor*		**			**		↓
F45D3.4	V	12552186	-		**			**		↑
*ugt-16*	V	12825004	UDP-glucuronosyl transferase	*			↓	**		↓
T16G1.4	V	12940017	Small molecule kinase*	*			↑	**		↑
F35E12.5	V	13737072	-	**			↓	**		↓
*cyp-35C1*	V	13898467	Cytochrome P450 family	**			↓	**		↓
*ret-1*	V	14829155	Reticulon protein					**		↓
*cdr-1*	V	15921335	Cadmium responsive	*			↓	**		↓
*nhr-127*	V	16262268	Nuclear hormone receptor family	**	**		↑	*		↑
T26H2.5	V	19230746	-	**			↑	*		↑
*daf-28*	V	19810833	Beta-type insulin	*	**		↓	**	**	↓
K02E2.7	V	20380157	-					**	**	↑
B0310.3	X	510900	-					**		↑
T10H10.2	X	2294743	FAD-dependent sulfhydryl oxidase/quiescin and related proteins*		**			**	**	↑
*tag-18*	X	3731903	-	*	**		↓	**	**	↓
*amt-1*	X	4571419	Ammonium transporter homologue	*	**	**	N2↑			
F13D11.3	X	5804348	-	**	**		↑			
*nnt-1*	X	6122273	Nicotinamide nucleotide transhydrogenase	*			↑	**		↑
C46F2.1	X	8077686	-					**		↓
D2021.8	X	8552200	-	**	**	**	↓			
C35C5.8	X	11533977	-	*			↑	**		↑
*cah-4*	X	13542493	Carbonic anhydrase	*			↑	**		↑
F23D12.3	X	14442540	-	**	**		↓			
*egrh-1*	X	14845856	Early growth factor response factor homologue					**		↑
F09B12.3	X	15095252	-	*			↑	**		↑

The 89 genes, their linkage group (LG) and location (Start), whose expression is significantly affected by TREATMENT (T), or TREATMENT × LINE (T × L) at *p *< 0.001 (**) and under dauer larva-inducing (DI) conditions up (↑) or down (→) regulation, and this specified for N2 or DR1350 specific TREATMENT × LINE effects. For these genes (above) other significant TREATMENT (*p *< 0.05 and *p *< 0.001) and LINE (L) (*p *< 0.001) only effects are also reported, with *p *< 0.05 and *p *< 0.001 shown by * and **, respectively. Gene is the WormBase gene identification [41], with NCBI KOGs shown by *.

#### Gene expression changes are consistent with metabolic changes

Analysis of these 89 genes showed that those up-regulated in dauer larva-inducing conditions were over-represented in mount 8 (Table 4). No specific biological function has been ascribed to these genes, but they do encompass many genes involved in digestion and intestinal function [37]. The genes down-regulated in dauer larva-inducing conditions were over-represented in lipid metabolism, cytochrome P450 and mount 19 (Table 4). This down-regulation of genes in mount 19 was consistent with previous observations in which these genes were comparatively down-regulated in adult wild-type, compared with *daf-16*, worms (*daf-16 *activity is required for dauer larva development) [6], as is the over-representation of genes in mount 8 [6]. Similarly, changes in the expression of cytochrome P450 genes have been found both on entry to and exit from the dauer larva stage [5,10]. Larvae that are destined to develop into dauer larvae have an altered metabolism, compared with non-dauer larva-destined forms [1], and these findings are therefore consistent with the initial stages of this move to a dauer larva-type metabolism.

**Table 4:** Gene lists and gene expression in different environments.

Gene list	Size	Genes	*p *value
**Down-regulated in dauer larva-inducing conditions**			

Lipid metabolism	300	C03G6.15	< 0.001
		C06B3.3	
		K07C6.5	
		ZC443.6	
		F10D2.11	
		C08F11.8	
Mount 19	185	F55G11.2	< 0.002
		ZK896.7	
		C03G6.15	
		K07C6.5	
		C14C6.5	
Cytochrome P450	40	C03G6.15	< 0.005
		06B3.3	
		K07C6.5	

**Up-regulated in dauer larva-inducing conditions**			

Mount 8	803	ZK550.6	< 0.001
		F45D3.4	
		T22F3.11	
		C35C5.7	
		DH11.2	
		C17H12.4	
		T16G1.4	
		K03H6.2	

Analysis of the 89 genes whose expression is significantly affected by exposure to non-dauer larva or dauer larva-inducing conditions. The gene lists that are significantly over represented among these 89 genes, Size is the number of genes within that list and Genes are the genes from the 89. *p *value is the probability that the occurrence of these genes in the gene list is random, with this Holm-Bonferroni corrected for multiple testing.

#### Over-representation of the DAF-16 binding site

Among these 89 genes (Figure 1, Table 3) the sequence CTTATCA occurred in 500 bp 5' to 43 (48%) of these genes. Across the whole *C. elegans *genome the CTTATCA sequence occurred within the same region in *c*.5% of genes. Therefore, this sequence is significantly over represented (*p *< 0.0001) among the putative regulatory regions of the 89 genes identified here. This sequence was previously identified as being over-represented upstream of genes whose expression differs between *daf-2(RNAi) *(wild-type lifespan) and *daf-16(RNAi); daf-2(RNAi) *(long lifespan) worms [12]. It has also been found to be over-represented upstream of those genes identified as DAF-16 targets by chromatin immunoprecipitation [14]. This sequence is therefore thought to represent either an alternative DAF-16 binding site or the binding site of an unknown gene that acts in combination with DAF-16 [12]. Further investigation of the possible role of DAF-16 in the regulation of the 89 genes identified here also indicated that the canonical DAF-16 binding sequence, TTGTTTAC [38], is present in the 500 bp 5' to 8 (9%) of these 89 genes. Further, the related sequence T(G/A)TTTAC, which is over-represented upstream of genes whose expression differs between *daf-2(RNAi) *(wild-type lifespan) and *daf-16(RNAi)*; *daf-2(RNAi) *(long lifespan) worms [12], was found in the 500 bp 5' to 23 (26%) of these 89 genes. This observation is also consistent with the observation that the starvation-induced arrest of L1 development is mediated by *daf-16 *[39]. Overall, these findings suggest that these genes may be the core transcriptional response of exposure to dauer or non-dauer larva-inducing conditions, and that this is at least in part controlled *via *the action of DAF-16.

#### Correspondence with QTLs affecting the plasticity of dauer larva development

Previously we have identified QTLs that control dauer larva formation under different environmental conditions and the plasticity of dauer larva development in N2 and DR1350 [17]. One of these is a *c*. 200 kbp region on chromosome II; this QTL does not encompass any loci previously identified to control dauer larva development [17]. Of the 89 genes (Table 3) whose expression is affected by dauer or non-dauer larva-inducing conditions one (F12E12.11) also occurs in this region and one other (Y110A2AL.4) is immediately adjacent to this region.

Overall, comparison of the QTL and expression analyses of the plasticity of dauer larva development show poor correspondence. This therefore suggests that these dauer larva development traits are genetically complex controlled, at least in part, by *trans*-acting loci. These *trans*-acting loci may be among the many loci that are differently transcribed between the lines.

## Conclusion

We have found that there are substantial differences between *C. elegans *lines in the transcriptional profile of mid-second stage larvae. These different profiles are commensurate with the likely other genomic differences between *C. elegans *lines. These transcriptional differences are concentrated in groups of putatively functionally-related genes, suggesting that each of these lines is following a different transcriptional profile. However, the gene expression data also suggest that these lines differ in their developmental rate. Therefore the apparent different transcriptional profiles, may also be a consequence of this different timing. Further, the different phenotypic plasticities of the lines may also be explained be different developmental rates, and hence inter-line differences in the 'window of sensitivity' to environmental change, of the lines.

We identified a small number of genes whose expression was altered in dauer or non-dauer larva-inducing conditions. Changed expression of so few genes is compatible with these early stage larvae commencing development as dauer or non-dauer larvae. We found evidence for a DAF-16 binding sequence upstream of many of these genes, suggesting that DAF-16 may co-ordinately control these changes in gene expression. These data, though, do not obviously expose the bases of the different phenotypic plasticities of dauer larva development among these four *C. elegans *lines. Comparison of these gene expression data with previous QTL data, reinforce that this phenotypic plasticity trait is genetically complex.

## Authors' contributions

SCH and MEV generated the RILs, AS prepared the larvae and RNA, GLAB analysed the hybridisation data with AS and SCH, SCH undertook further analysis and co-wrote the manuscript with MEV. MEV conceived and directed the study. All of the authors read and approved the final manuscript.

## Supplementary Material

Additional file 1 **Inter-line differences in gene expression**. The genes whose expression is significantly affected by LINE (*p *< 0.001), in experiment one (N2 and DR1350) and experiment two (RIL-14 and RIL-17) showing the Affymetrix identifier and annotation, the ANOVA *p *values for LINE, TREATMENT and LINE × TREATMENT, effects and the mean hybridisation intensities for DR1350 and N2 and for RIL-14 and RIL1-7, under non-dauer and dauer-inducing conditions and the direction of the N2 and DR1350 and the RIL-14 and RIL-17 difference. Click here for file
